# Influence of Physical Activity during Pregnancy on Maternal Hypertensive Disorders: A Systematic Review and Meta-Analysis of Randomized Controlled Trials

**DOI:** 10.3390/jpm14010010

**Published:** 2023-12-21

**Authors:** Rubén Barakat, Cristina Silva-Jose, Dingfeng Zhang, Miguel Sánchez-Polán, Ignacio Refoyo, Rocío Montejo

**Affiliations:** 1 AFIPE Research Group, Faculty of Physical Activity and Sport Sciences-INEF, Universidad Politécnica de Madrid, 28040 Madrid, Spain; cristina.silva.jose@upm.es (C.S.-J.); dingfeng.zhang@alumnos.upm.es (D.Z.); miguel.sanchez.polan@upm.es (M.S.-P.); 2 Faculty of Physical Activity and Sport Sciences-INEF, Universidad Politécnica de Madrid, 28040 Madrid, Spain; ignacio.refoyo@upm.es; 3 Department of Obstetrics and Gynecology, Institute of Clinical Sciences, Sahlgrenska Academy, University of Gothenburg, 41346 Gothenburg, Sweden; rocio.montejo.rodriguez@gu.se; 4 Department of Obstetrics and Gynecology, Sahlgrenska University Hospital, 41346 Gothenburg, Sweden

**Keywords:** pregnancy, physical activity, gestational hypertension, hypertensive disorders, preeclampsia, maternal health

## Abstract

Gestational hypertension is a notable concern with ramifications for maternal and fetal health. Preemptive measures, including physical activity (PA), are crucial. There is a pressing need for comprehensive investigations into the impact of various forms of PA on hypertensive disorders. A systematic review and meta-analysis (CRD42022372468) following the Preferred Reporting Items for Systematic Reviews and Meta-Analyses (PRISMA) guidelines was performed. Our review exclusively considered randomized clinical trials (RCTs) between 2010 and 2023, using the following databases: EBSCO, including Academic Search Premier, Education Resources Information Center, PubMed/MEDLINE, SPORTDiscus, and OpenDissertations; Clinicaltrials.gov; Web of Science; Scopus; the Cochrane Database of Systematic Reviews; and the Physiotherapy Evidence Database (PEDro). The primary outcome was hypertensive disorders occurring during pregnancy (14 studies). Diagnosed preeclampsia (15 studies) and blood pressure levels were also examined (17 studies). PA during pregnancy was significantly associated with a reduced risk of hypertensive disorders (RR = 0.44, 95% CI = 0.30, 0.66). The data also indicate a positive correlation between PA during pregnancy and both systolic (MD = −2.64, 95% CI = −4.79, −0.49) and diastolic (MD = −1.99, 95% CI = −3.68, −0.29) blood pressure levels. The relationship between PA and the incidence of diagnosed preeclampsia did not demonstrate a statistically significant association (RR = 0.81, 95% CI = 0.59, 1.11; *p* = 0.20). Random effects were used for all analyses. PA during pregnancy promises to improve maternal health by reducing the risk of gestational hypertension and positively affecting systolic and diastolic blood pressure.

## 1. Introduction

Epigenetics has profoundly revolutionized the comprehension and analysis of numerous essential processes, with pregnancy standing out as one of the domains most profoundly influenced by this paradigm shift. It underscores the pivotal role of the intrauterine environment as a determining factor for the offspring’s lifelong health trajectory. Consequently, maintaining a state of equilibrium within various domains of the female organism (including cardiovascular, metabolic, and emotional aspects) during gestation emerges as a linchpin for ensuring the prospective well-being of the developing human [1].

Conversely, complications and pathologies arising during pregnancy wield the potential to impose enduring health ramifications, some of which might manifest chronically over time. The wealth of the scientific literature substantiates these assertions, firmly grounding them within empirical evidence [2]. Within this context, the journey of pregnancy and childbirth stands unparalleled in its transformative nature, given the substantial quantitative and qualitative metamorphoses that the female body must undergo to facilitate optimal fetal growth and development. Moreover, the female physique must adeptly navigate the critical juncture of delivery, orchestrating the efficient expulsion of the fetus into the extracorporeal milieu while upholding the proficiency of its multifaceted systems [3].

Foremost among these systems, the cardiovascular apparatus assumes paramount significance, being subjected to heightened demands throughout gestation and particularly during delivery. Over 40 weeks, the cardiovascular system of the pregnant woman undergoes structural and functional adaptations spurred by two fundamental factors: escalating requisites due to ongoing fetal development and the mechanical consequence of the enlarging uterus causing the repositioning of specific structures. Mastery of these modifications and preserving a harmonious maternal–fetal equilibrium, with a vigilant eye on averting cardiovascular complications and pathologies, stand as formidable challenges confronting the pregnant woman [4].

Extensive research signals the perils of an imbalanced cardiovascular milieu during pregnancy. Gestational hypertension is characterized by elevated blood pressure levels that manifest specifically during pregnancy. Diagnosis typically involves the identifying two consecutive elevated blood pressure readings equal to or exceeding 140/90 mmHg (classified within the mild range) after the 20th week of gestation, with the readings taken at least 4 h apart. Preeclampsia is a pregnancy-related condition characterized by high blood pressure and signs of damage to organ systems, often the liver and kidneys. Preeclampsia is defined as newly elevated blood pressure ≥140/90 after 20 weeks of gestation in addition to proteinuria, defined as 300 mg or more in a 24-h urine specimen, or a protein/creatinine ratio of 0.3 or more, or 2+ protein on urine dipstick (used if other quantitative methods are unavailable). Eclampsia is a severe complication of preeclampsia, characterized by seizures in pregnant women. HELLP syndrome involves hemolysis, elevated liver enzymes, and low platelet count, further escalating the risks associated with preeclampsia. Notably, gestational hypertension emerges as a conceivable complication, the repercussions of which reverberate across maternal, fetal, and neonatal domains, wielding lasting ramifications for the subsequent well-being of the individual [5,6].

Graver aberrations such as preeclampsia, elusive to effective control, engender pregnancies fraught with intensive medical monitoring and even imperil fetal viability. The imperative of preemptive measures, operative across the gestational timeline, becomes evident in this intricate landscape. Among these measures, moderate physical activity emerges as a promising prophylactic agent, yielding favorable outcomes across various facets of the female anatomy. Particular attention should be given to mitigating metabolic imbalances, notably excess maternal weight gain, and addressing the epigenetic repercussions of an adverse intrauterine environment [1,2,3,7].

The World Health Organization recommends at least 150 min of moderate-intensity aerobic activity spread throughout the week for pregnant women, barring contraindications. This promotes overall health and may reduce the risk of gestational hypertension [8].

Numerous studies have investigated the influence of physical activity (PA) on gestational hypertension. However, the findings have been inconclusive, lacking a definitive conclusion. In this context, implementing new research methodologies characterized by high reliability and rigor, such as a systematic review and meta-analysis (SR + MA), can significantly contribute to advancing scientific knowledge in this area.

We hypothesize that engaging in physical activity during pregnancy may serve as a preventive factor against hypertensive disorders and associated complications in pregnant women.

This systematic review and meta-analysis (SR+MA) aimed to examine the effects of diverse modalities of PA during pregnancy on the prevention of gestational hypertension.

## 2. Materials and Methods

The Preferred Reporting Items for Systematic Reviews and Meta-Analyses (PRISMA) guidelines were followed in the current study. It was registered with the International Prospective Register of Systematic Reviews (PROSPERO, Registration No. CRD42022372468). The Population, Intervention, Comparison, Outcomes, and Study Design (PICOS) framework was used to analyze the search sources [9].

### 2.1. Population

The population included pregnant individuals without obstetric contraindications to exercise during pregnancy who participated in a prenatal PA program [10].

### 2.2. Intervention

The analyzed characteristics of the intervention were as follows: (a) weekly frequency of PA sessions; (b) intensity, where all included studies utilized a moderate load intensity, defined as 55–65% of the maximum maternal heart rate or the perceived effort on the Borg Scale (range 12–14); (c) duration of the PA program; (d) type of PA, including yoga, Pilates, aerobic exercises, strength training, or pelvic floor training; (e) supervision of the PA program; and (f) duration of the individual sessions, as presented in Table 1.

### 2.3. Comparison

Since all the studies analyzed were randomized clinical trials (RCTs), women who participated in an exercise or PA program during pregnancy were compared with those who did not. Intervention characteristics were collected and compared, as presented in Table 1.

### 2.4. Outcomes

The primary outcome was hypertensive disorders occurring during pregnancy. Diagnosed preeclampsia and blood pressure levels were also examined.

### 2.5. Study Design and Selection Process

As previously mentioned, all eligible articles were RCTs. The literature search was conducted between September 2022 and May 2023 at Universidad Politécnica de Madrid (INEF), using the following databases: EBSCO, including Academic Search Premier, Education Resources Information Center, PubMed/MEDLINE, SPORTDiscus, and OpenDissertations; Clinicaltrials.gov; Web of Science; Scopus; the Cochrane Database of Systematic Reviews; and the Physiotherapy Evidence Database (PEDro). The search encompassed articles written in English or Spanish that were published between 2010 and 31 May 2023. The search terms used were:

English: (“physical activity” OR “exercise” OR “training” OR “physical exercise” OR “fitness” OR strength training” OR “physical intervention” OR “maternal exercises” OR “cointervention”) AND (“pilates” OR “yoga” OR “strengthening” OR aerobic OR “resistance training” OR “walking”) AND (“pregnancy” OR “maternal” OR “antenatal” OR “pregnant” OR “gestation”) AND (“health” OR “wellbeing”) AND (“randomized clinical trial” OR “RCT” AND (“hypertensive disease” OR “hypertensive disorders” OR “hypertension”) AND (“blood pressure”) AND (“preeclampsia”) AND (“Systolic” AND “diastolic”).

Spanish: (“actividad física” OR “ejercicio” OR “entrenamiento” OR “ejercicio físico” OR “fitness” OR entrenamiento de fuerza” OR “intervención física” OR “ejercicios maternos” OR “cointervención”) AND (“pilates” OR “yoga” OR “fortalecimiento” OR “aeróbico” OR “entrenamiento de resistencia” OR “caminar”) AND (“embarazo” OR “materno” OR “prenatal” OR “embarazada” OR “gestación”) AND (“salud” OR “bienestar”) AND (“ensayo clínico aleatorizado” OR “ECA” AND (“enfermedad hipertensiva” OR “trastornos hipertensivos” OR “hipertensión”) OR (“presión arterial”) OR (“preeclampsia”) OR (“Sistólica y Diastólica”).

**Figure 1:**
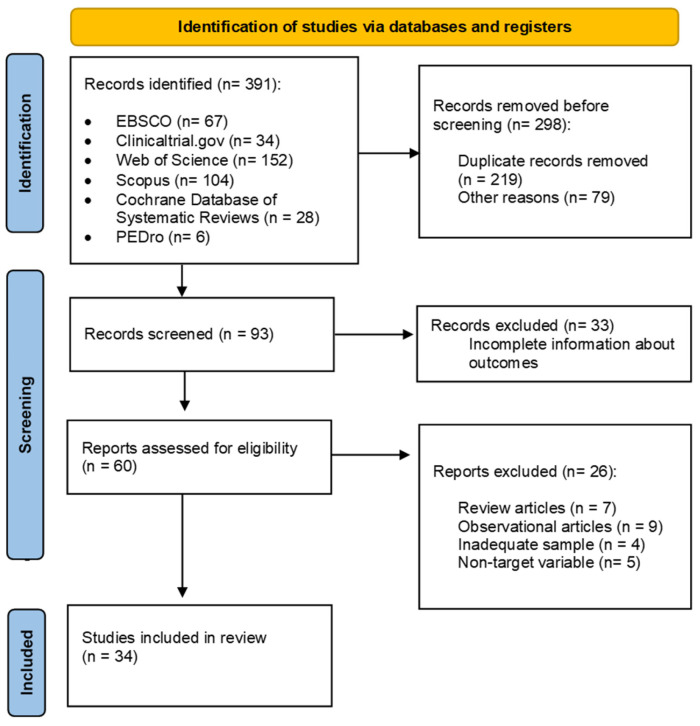
Flow diagram of the analyzed articles [11,12,13,14,15,16,17,18,19,20,21,22,23,24,25,26,27,28,29,30,31,32,33,34,35,36,37,38,39,40,41,42,45,46].

The eligible articles for review comprised studies that measured PA or exercise intervention (excluding articles that solely provided advice for an active pregnancy or those that included a measurable PA questionnaire but lacked an exercise intervention). The characteristics of the PA or exercise program were also considered. The protocol for screening records and extracting data was performed by two researchers (D.Z. and M S-P). This process is illustrated in Figure 1. Two reviewers (R.B. and C.S.-J.) independently screened titles and abstracts of all identified citations, and potentially eligible articles were selected. Full-text articles were independently assessed by the two reviewers for eligibility criteria. Any discrepancies were resolved by consensus.

Additionally, secondary outcomes such as physiological, sociodemographic, and delivery outcomes were examined by two reviewers (D.Z. and M.S.-P.) to evaluate the effects of each intervention on maternal health. However, these secondary outcomes were not included in the meta-analyses. From each selected study, we extracted the following information: author(s), publication year, country where the study was conducted, study design type, number of participants, characteristics of the intervention program, and the variables analyzed (both primary and secondary outcomes).

### 2.6. Statistical Analysis, Quality of Evidence Assessment, Risk of Bias, and Publication Bias

Meta-analyses were conducted, with the dependent variable being the ratio of occurrence of hypertensive disorders in each study, categorized as either “yes” or “no”. The number of events observed in each study group and their respective relative risks (RRs) were recorded. A random effects model calculated the total risk ratio (RR) sum [47]. Each study was assigned a weight based on its sample size, contributing to the overall analysis, and establishing a weighted average. The I^2^ statistic was utilized to quantify the heterogeneity observed in the results due to variations in interventions and study designs, indicating the extent of variability in the effects of each intervention, which were non-random. The following criteria were used to classify heterogeneity levels: low heterogeneity (25%), moderate heterogeneity (50%), and high heterogeneity (75%) [48].

The quality of evidence for each study’s primary outcome was assessed using the Grading of Recommendations Assessment, Development, and Evaluation (GRADE) framework, with RCT studies rated as moderate or high quality. The certainty of evidence was high [49]. The Cochrane Handbook guidelines were followed to evaluate the potential risk of bias (including selection, performance, attrition, detection, and reporting bias) [45]. RCTs were initially considered to have a “low” risk of bias due to their study design and intervention compared with non-randomized interventions. However, their risk of bias could be either increased or decreased depending on the presence of “high” or “low” scores across the different bias sources [49].

In order to assess potential publication bias in each developed meta-analysis, the Egger regression test was used due to its enhanced sensitivity in detecting publication bias under conditions of weak or moderate heterogeneity. Typically, this test yields a metric indicating significant publication bias when *p* < 0.1 [46].

## 3. Results

### 3.1. Study Selection

A total of 391 articles were initially identified during the first stage of the search of which 298 were removed before screening: duplicate records removed (n = 219) and other reasons (n = 79) such as they did not meet the inclusion criteria. After record screening, 33 articles were excluded. Subsequently, 26 articles were excluded for the following reasons: being review (n = 7) or observational (n = 9) studies, inadequate sample (n = 4), and non–target variable (n = 5). Ultimately, 34 studies were included for further meta-analysis (Figure 1).

### 3.2. Risk of Bias Assessment

**Figure 2:**
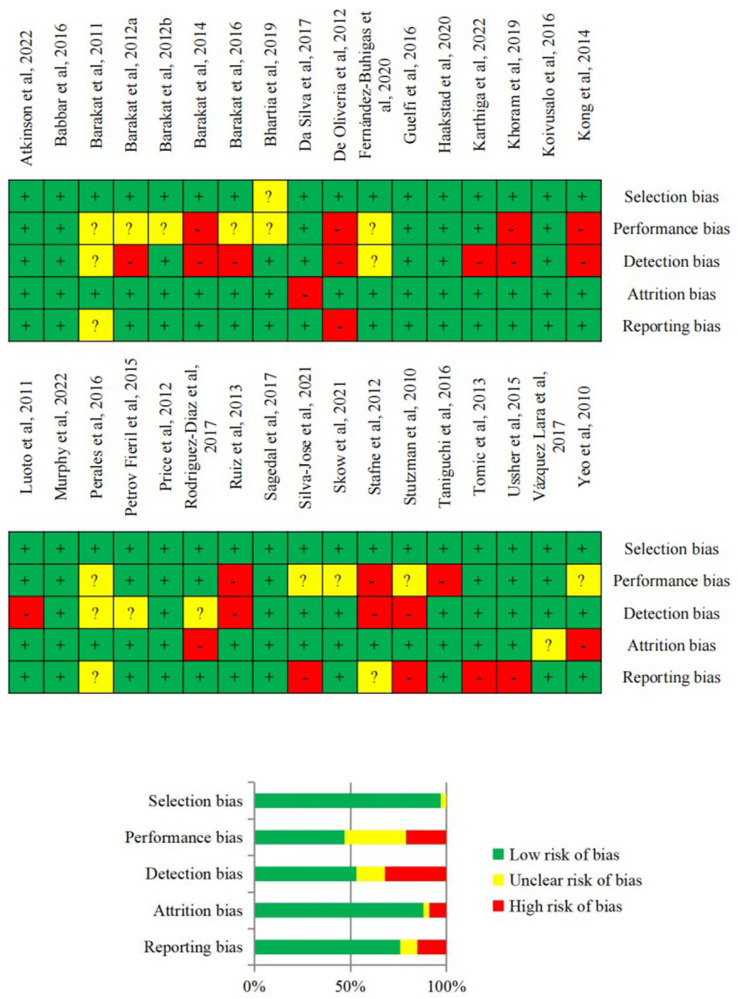
Risk of bias assessment [16,17,18,19,20,21,22,23,24,25,26,27,28,29,30,31,32,33,34,35,36,37,38,39,40,41,42,43,44,45,46,47,48,49].

The quality of evidence across the studies exhibited a spectrum ranging from low to high. Achieving blinding of participants to either the intervention or control group, as well as blinding of the instructor, was often unattainable due to the inherent characteristics of the PA intervention. Consequently, this led to an unclear or high risk of bias, particularly with regard to performance bias, depending on how it was documented. In some instances, other potential sources of bias included the unavailability of the study protocol for public scrutiny, preventing a comparison between the planned and observed outcomes. Additionally, there were instances of a need for more transparency in reporting the randomization process. However, on the whole, the majority of the studies demonstrated a low risk of bias across the five categories of bias that were evaluated. The detailed risk of bias analysis can be found in Figure 2.

### 3.3. Effect of PA on the Occurrence of Hypertensive Disorders

**Figure 3:**
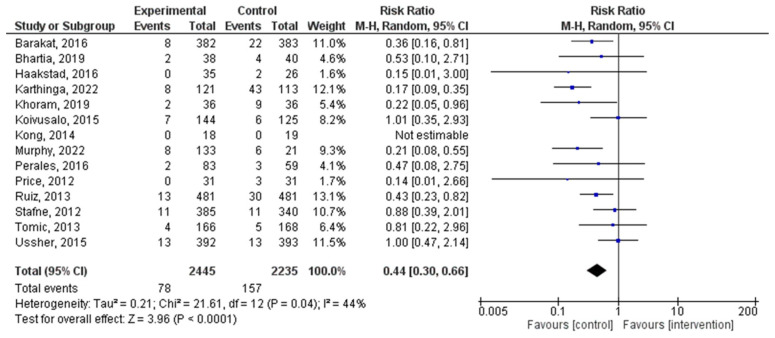
Effect of PA on the occurrence of hypertensive disorders [22,23,28,29,30,31,32,34,35,37,39,46,47].

A total of 14 distinct RCTs were incorporated into the present analysis, which investigated the incidence of hypertensive disorders in women within both experimental and control groups during pregnancy. The results revealed a significant correlation between PA during pregnancy and the occurrence of hypertensive disorders (RR = 0.44, 95% CI = 0.30, 0.66, *p* = 0.0002; I^2^ = 53%, P_heterogeneity_ = 0.01). Figure 3 depicts a forest plot corresponding to the conducted meta-analysis. The quantification evaluation of the risk of publication bias test in the analyzed articles showed that there was no potential publication bias (*p* = 0.44) in this analysis.

### 3.4. Effect of PA during Pregnancy on Diagnosed Preeclampsia

**Figure 4:**
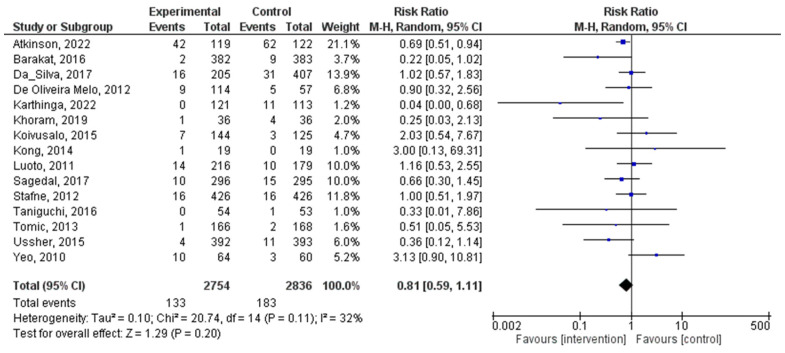
Effect of PA during pregnancy on diagnosed preeclampsia [16,22,24,25,29,30,31,32,33,40,43,45,46,47,49].

A total of 15 distinct RCTs were included in the present analysis, which examined the incidence of diagnosed preeclampsia in women within both experimental and control groups during pregnancy. The results did not reveal a significant statistical association between PA during pregnancy and the diagnosed preeclampsia (RR = 0.81, 95% CI = 0.59, 1.11; I^2^ = 26%, P_heterogeneity_ = 0.18). Figure 4 illustrates a forest plot corresponding to the conducted meta-analysis. The quantitative assessment of publication bias risk in the analyzed articles indicated the absence of potential publication bias (*p* = 0.99) in this analysis.

### 3.5. Effect of PA during Pregnancy on Systolic Blood Pressure

**Figure 5:**
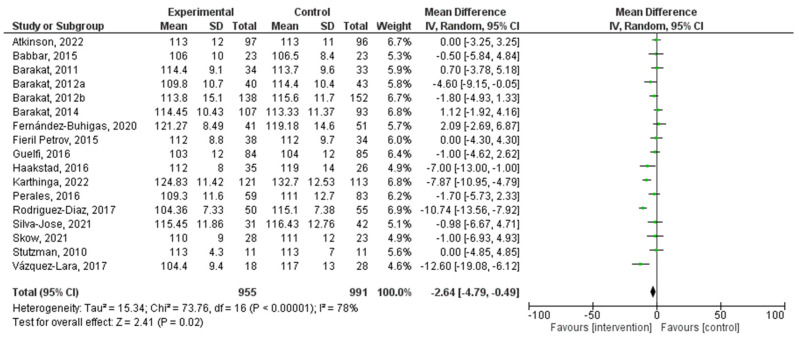
Effect of PA during pregnancy on systolic blood pressure [16,17,18,19,20,21,26,27,28,29,35,36,38,41,42,44,48].

There was a total of 17 studies that were incorporated into this analysis. Regular PA during pregnancy had a significant relationship with systolic blood pressure (MD = −2.64, 95% CI = −4.79, −0.49, I^2^ = 78%, P_heterogeneity_ = 0.00001). The forest plot corresponding to the current meta-analysis is illustrated in Figure 5. The quantification evaluation of the risk of publication bias test in the analyzed articles showed that there was no potential publication bias (*p* = 0.8) in this analysis.

### 3.6. Effect of PA during Pregnancy on Diastolic Blood Pressure

**Figure 6:**
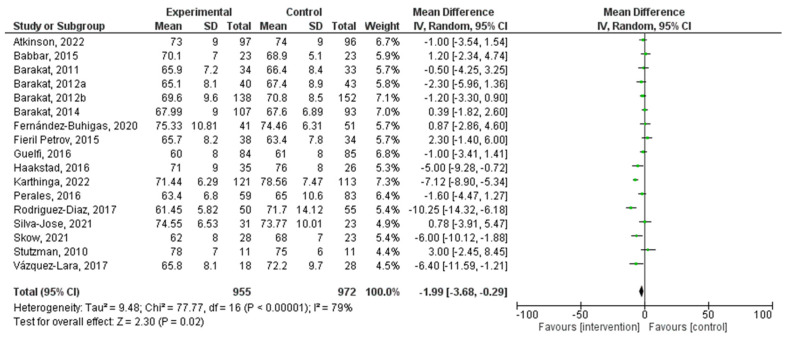
Effect of PA during pregnancy on diastolic blood pressure [16,17,18,19,20,21,26,27,28,29,35,36,38,41,42,44,48].

There was a total of 17 studies that were incorporated into this analysis. Regular exercise or PA during pregnancy had a significant relationship with diastolic blood pressure (MD = −1.99, 95% CI = −3.68, −0.29, I^2^ = 79%, P_heterogeneity_ = 0.00001). The forest plot corresponding to the current meta-analysis is illustrated in Figure 6. The quantitative assessment of publication bias risk in the analyzed articles indicated the absence of potential publication bias (*p* = 0.63) in this analysis.

## 4. Discussion

In light of the current study’s findings, it is evident that active women during pregnancy exhibit a noteworthy decrease in hypertensive disorders. Additionally, a favorable impact of physical activity during pregnancy on both systolic and diastolic blood pressure is discernible. These results provide valuable insights into the potential benefits of exercise for expectant mothers and their overall maternal health, supporting international recommendations for a minimum amount of moderate PA during pregnancy (150 min per week) as a facilitator of positive effects of PA during pregnancy on maternal, fetal, and newborn outcomes [8,10,50].

The rigorous study selection aimed to ensure the inclusion of only high-quality research in this meta-analysis. This careful selection process enhances the reliability and validity of the meta-analysis results.

The mechanism underlying the beneficial impact of moderate exercise on blood pressure in pregnant women is a multifaceted interplay of physiological adaptations that contribute to cardiovascular health. Several studies have indicated that engaging in regular, moderate-intensity physical activity during pregnancy can improve cardiac function, regulate body weight, reduce oxidative stress, and enhance vascular endothelial function. The type and intensity of exercise should be adapted to the woman’s pre-existing fitness level. Aerobic exercises, such as those included in our meta-analysis, have been associated with increased cardiac output and stroke volume. These cardiovascular adaptations play a crucial role in maintaining optimal blood flow to vital organs, including the placenta, supporting the overall well-being of both the mother and the developing fetus [51,52].

Moreover, the positive effects of moderate exercise extend to its impact on body weight, which is a significant factor in blood pressure regulation. Exercise during pregnancy has been shown to contribute to healthy weight management, reducing the risk of excessive gestational weight gain, a known factor associated with elevated blood pressure [53].

Oxidative stress, implicated in the pathogenesis of hypertensive disorders, is another aspect influenced by moderate exercise. Regular physical activity has been linked to reducing oxidative stress markers and promoting an environment conducive to blood pressure homeostasis [54].

Additionally, improvements in vascular endothelial function, facilitated by exercise, contribute to the dilation of blood vessels, enhancing blood flow and reducing the resistance that can lead to elevated blood pressure. The adaptability of the vascular system is particularly crucial during pregnancy, where adequate blood supply is essential for the developing fetus [55].

One notable aspect of the included studies was the diverse nature of the interventions. These interventions were delivered by specialized professionals and encompassed various physical activities, including aerobic exercises, strength training, and aquatic sessions. The variety in exercise modalities reflects the versatility of PA programs during pregnancy. Furthermore, the session frequency and duration flexibility allow interventions tailored to individual preferences and needs. Compared with previous studies in the field, this meta-analysis stands out due to its focused examination of specific maternal health outcomes, including the latest evidence, and its interpretation of clinical significance. While previous studies may have explored broader pregnancy-related outcomes, this study homes in on hypertensive disorders, preeclampsia, and blood pressure levels, providing a more targeted analysis.

The meta-analysis identified a significant correlation between exercise during pregnancy and the occurrence of hypertensive disorders. This finding suggests that regular PA may protect against developing hypertensive disorders in pregnant women. The RR of 0.44, with a 95% confidence interval, indicates a substantial reduction in the risk of hypertensive disorders among pregnant women participating in PA programs. However, it is essential to note that a moderate level of heterogeneity (I^2^ = 53%) was observed among the included studies, suggesting some variability in the results. Further research may help elucidate the sources of this heterogeneity. Our findings align with the results of the Magro-Maloso et al. 2017 and Martinez-Vizcaino et al. 2022 meta-analyses [56,57], which also assessed the impact of aerobic exercise during singleton pregnancies on hypertensive disorders. While our study includes additional evidence and focuses on specific outcomes, both analyses support the notion that exercise during pregnancy is associated with a reduced risk of gestational hypertensive disorders.

Additionally, our meta-analysis and Wolferz et al.’s 2017 systematic review harmonize the exploration of aerobic exercise’s impact on gestational hypertensive disorders [58]. Both studies find that engaging in regular aerobic exercise, particularly starting early in pregnancy, holds promise in reducing the risk of these disorders. This work also emphasized that the type of exercise, specifically aerobic exercise, significantly reduced the risk of gestational hypertensive disorders. This concept resonates with our findings. While our study did not specifically investigate the impact of exercise on preterm birth rates, Wolferz et al.’s 2017 systematic review suggests that exercise during pregnancy may not influence preterm birth rates [58]. Our meta-analysis, which focused on exercise interventions during pregnancy, echoes the findings of Zhang et al.’s 2023 study [59]. While their article provides insights into the multifaceted pathogenesis of gestational hypertension, our research delves into exercise’s potential preventive and therapeutic role in addressing this complex condition. The physiological mechanisms discussed in Zhang et al.’s work, such as improvements in cardiac function, body weight, oxidative stress reduction, and vascular endothelial function with exercise, align with our findings regarding the positive effects of exercise on blood pressure regulation during pregnancy.

In contrast to the positive association with hypertensive disorders, this meta-analysis did not reveal a statistically significant relationship between exercise during pregnancy and the incidence of diagnosed preeclampsia. The RR of 0.81, with a 95% confidence interval, suggests that exercise does not substantially impact the development of preeclampsia. However, it is worth noting that there was a relatively low level of heterogeneity (I^2^ = 18%) among the studies, indicating a degree of consistency in the results. While exercise may not significantly reduce the risk of preeclampsia, it is essential to consider the potential benefits of PA on other aspects of maternal health. In contrast to the findings of Davenport et al. 2017 [60], our study did not observe a significant reduction in preeclampsia (PE) risk with exercise interventions during pregnancy. Magro-Malosso et al. 2017 did not find a significant difference in preeclampsia incidence between exercise and control groups, likely due to an underpowered analysis requiring a larger sample size to detect a 21% reduction in preeclampsia from a 2.3% baseline risk [56]. A Cochrane Review from 2006 and prior meta-analyses also support the positive effects of exercise on maternal health outcomes, including reductions in gestational diabetes and maternal hypertension. However, no significant impact on preeclampsia was observed [61].

Several hypotheses may explain why exercise during pregnancy did not show substantial benefits for preeclampsia in the meta-analysis as preeclampsia is a complex pathology. While beneficial for cardiovascular health, exercise may not directly address the intricate mechanisms leading to preeclampsia. Other factors, such as genetic predisposition and immunological factors, might play a more dominant role in the development of preeclampsia. The timing and intensity of exercise during pregnancy could be other critical factors. The exercise regimens in the included studies did not match the specific timing or dose required to prevent preeclampsia. Preeclampsia often develops in the later stages of pregnancy, and the impact of exercise may vary depending on when it is initiated and its intensity. Pregnant women are a diverse group, and individual responses to exercise can differ significantly. Some women may have genetic or physiological factors predisposing them to preeclampsia regardless of exercise. The included studies did not adequately consider this variability, which may have reduced the benefits and should be planned for future studies on this scientific topic.

The analysis of systolic blood pressure levels revealed a significant association with regular exercise during pregnancy. A mean difference of −2.64 (95% CI = −4.79, −0.49) suggests a modest reduction in systolic blood pressure among pregnant women who participate in PA. However, it is essential to note that a relatively high level of heterogeneity (I^2^ = 76%) was observed in this analysis. This heterogeneity may be attributed to variations in exercise intensity, frequency, and duration variations among the included studies. Despite the heterogeneity, the observed decrease in systolic blood pressure aligns with the overarching objective of enhancing maternal health during pregnancy.

Similarly, the analysis of diastolic blood pressure also revealed a significant difference (MD = −1.99, 95% CI = −3.68, −0.29). Similar to systolic blood pressure, this analysis exhibited a high level of heterogeneity (I^2^ = 81%). This heterogeneity underscores the intricacies of studying blood pressure responses to exercise during pregnancy, as multiple factors can influence blood pressure outcomes, including complex dynamics, individual variations, and measurement precision. Exercise appears to positively impact on both systolic and diastolic blood pressure, despite the challenges posed by the complex nature of studying blood pressure dynamics in pregnant individuals. An understanding of these complexities can guide future research and healthcare recommendations for pregnant women seeking to enhance their cardiovascular health through exercise.

One of the key strengths of this study is its incorporation of the most recent RCTs and research available up to the present date. This study acknowledges and quantifies heterogeneity among the included studies, demonstrating a commitment to rigorous analysis. This transparency enhances the robustness and reliability of the findings, promoting greater confidence in the results. This study’s exploration of the null findings related to preeclampsia is a noteworthy strength. By offering hypotheses and insights into why exercise may not have demonstrated significant benefits for this specific condition, this study contributes to a more comprehensive understanding of the intricate relationship between exercise and maternal health. While this study transparently addresses heterogeneity, it remains a challenge inherent to meta-analyses. Variations in study design, interventions, and populations may contribute to heterogeneity, potentially affecting the generalizability of the findings. This study’s findings are based on the available evidence, which may predominantly represent specific populations or settings. As such, the generalizability of the results to broader people or diverse healthcare contexts may be limited. Future studies may need to fully account for variability in participants’ adherence and compliance with exercise interventions. Variations in adherence could impact the effectiveness of exercise programs and influence the outcomes observed in meta-analyses. The absence of significant results in some studies may introduce publication bias, as studies demonstrating no effect of exercise on maternal health outcomes may be less likely to be published or included in meta-analyses. This bias could influence the overall findings.

In the context of sports medicine, our meta-analysis offers valuable insights into the effects of PA during pregnancy on hypertensive disorders, preeclampsia, and blood pressure levels. These findings are significant for expectant mothers’ overall maternal health. By carefully selecting 34 studies from an initial pool of 391 articles, we aimed to ensure the inclusion of high-quality research, thus enhancing the credibility of our results. Notably, the diversity of interventions in the included studies, ranging from aerobic exercises to strength training and aquatic sessions, reflects the adaptability of pregnancy-related PA programs.

Our meta-analysis distinguishes itself by focusing on specific maternal health outcomes and emphasizing clinical significance, in contrast to previous broader studies. By integrating the latest research and addressing heterogeneity, we offer a clear perspective on the study variations. We found a substantial correlation between exercise during pregnancy and reduced hypertensive disorder risk, while preeclampsia risk reduction was inconclusive. Furthermore, exercise demonstrated a modest reduction in systolic blood pressure levels. These insights inform the complex relationship between exercise and maternal health, offering guidance for future research and healthcare recommendations.

## 5. Conclusions

PA during pregnancy promises to improve maternal health by reducing the risk of gestational hypertension and positively affecting systolic and diastolic blood pressure. In conclusion, our meta-analysis underscores the positive impact of PA during pregnancy on maternal health, specifically in reducing the risk of gestational hypertension and positively influencing systolic and diastolic blood pressure. The mechanisms underlying these benefits involve improvements in cardiac function, body weight regulation, oxidative stress reduction, and vascular endothelial function. Engaging in aerobic exercise and moderate physical activity three times per week appears to be the most suitable regimen throughout pregnancy. However, addressing the broader questions raised, the optimal frequency, duration, and type of exercise for pregnant women remain nuanced and should be tailored to individual preferences and needs.

## Figures and Tables

**Table 1:** Characteristics of analyzed articles.

Author	Year	Country	N	EG	CG	Intervention: Physical Exercise Program							Main Variables Analyzed	Secondary Variables Analyzed
						Freq	Intensity	Duration of Program	Type of Exercise	Superv. Classes	Duration of Class	Adh.		
Atkinson [11]	2022	Canada	241	119	122	3–4	Mod	22 w	Walking	No	25–40 min	80%	Gestational weight gain	Hypertension, type of delivery and birth weight
Babbar [12]	2016	USA	46	23	23	3	Mod	8 w	Yoga	Yes	60 min	80%	Umbilical artery, type of delivery, birth weight	Gestational weight gain, hypertension
Barakat [13]	2011	Spain	80	40	40	3	Low–Mod	28 w	Aerobic and light strength, PFMT	Yes	35–45 min	90%	Maternal health status, urinary incontinence	Gestational age, type of delivery, hypertension
Barakat [14]	2012	Spain	83	40	43	3	Low–Mod	28 w	Land aerobic and aquatic activity	Yes	35–45 min	-	Gestational weight gain and gestational diabetes	Type of delivery, birth weight, hypertension
Barakat [15]	2012	Spain	290	138	152	3	Mod	28 w	Aerobic exercise	Yes	40–45 min	-	Type of delivery	Gestational weight gain, birth weight, hypertension
Barakat [16]	2014	Spain	200	107	93	3	Low–Mod	28 w	Aerobic exercise, PFMT	Yes	55–60 min	80%	Gestational weight gain, type of delivery, hypertension, gestational diabetes	Birth weight, head circumference
Barakat [17]	2016	Spain	765	382	383	3	Mod	28 w	Aerobic, strength, and flexibility exercise	Yes	50–55 min	80%	Hypertension, macrosomia	Type of delivery, gestational weight gain, birth weight
Bhartia [18]	2019	India	78	38	40	1	Mod	12 w	Yoga	Yes	50 min	-	Maternal stress, type of delivery, birth weight	Hypertension
						2				No				
da Silva [19]	2017	Brazil	639	213	426	3	Mod	16 w	Aerobic, strength training	Yes	60 min	70%	Preterm birth, preeclampsia, hypertension	Gestational weight gain, birth weight
de Oliveria [20]	2012	Brazil	111	54	57	3	60–80% Max HR	25 w	Walking	Yes	15–40 min	85%	VO2max, birth weight and gestational age	Hypertension
Fernández-Buhigas [21]	2020	Spain	92	41	51	3	50–60% Max HR	28 w	Aerobic and light strength, PFMT	Yes	60 min	70%	Hypertension, metabolic, hepatic	Renal
Guelfi [22]	2016	Australia	172	85	87	3	Mod	14 w	Home-based stationary cycling program	Yes	20–60 min	-	Gestational diabetes	Type of delivery, birth weight, hypertension
Haakstad [23]	2020	Norway	90	43	47	2	Mod	12 w	Aerobic, PFMT	Yes	60 min	80%	Duration of labor, type of delivery, episiotomy	-
						3				No	30 min			
Karthiga [24]	2022	India	234	121	113	7	Mod	20 w	Yoga Five sessions of yoga techniques	No	60 min	-	Gestational hypertension	Type of delivery, duration of labor, birth weight
Khoram [25]	2019	Iran	74	38	36	4	Mod	20 w	Walking	No	20–30 min	-	Gestational hypertension	Preeclampsia
Koivusalo [26]	2016	Finland	293	155	138	5	Mod	22 w	Aerobic, dietary counseling	No	30 min	-	Gestational diabetes	Hypertension
Kong [27]	2014	USA	37	18	19	5	Mod	22 w	Walking	No	30 min	-	BMI, gestational weight gain	Hypertension
Luoto [28]	2011	Finland	399	219	180	7	Mod	27 w	Aerobic, dietary counseling	No	30 min	-	Gestational diabetes, gestational age	Hypertension
Murphy [29]	2022	USA	154	63	21	3	Mod	20 w	Aerobic	Yes	50 min	-	Hypertension	Heart rate
				33					Resistance					
				37					Combination					
Perales [30]	2016	Spain	241	120	121	3	Light Mod	28 w	Aerobic and strength exercises	Yes	55–60 min	-	Gestational weight gain, depression, hypertension	Duration of labor, type of delivery, birth weight,
Petrov Fieril [31]	2015	Sweden	72	38	34	2	Mod	12 w	Resistance training	Yes	60 min	-	Birth weight, gestational age	Hypertension, miscarriage
Price [32]	2012	USA	62	31	31	4	Mod	23 w	Aerobic exercise	Yes	45–60 min	-	Birth weight	Duration of labor, type of delivery, hypertension
Rodriguez-Diaz [33]	2017	Spain	100	50	50	2	Mod	8 w	Pilates	Yes	40–45 min	90%	Gestational weight gain, hypertension, strength, flexibility and spinal curvature	Type of delivery, episiotomy, analgesia, and birth weight
Ruiz [34]	2013	Spain	962	481	481	3	Light mod	28 w	Aerobic and resistance exercises	Yes	50–55 min	97%	Gestational weight gain	Hypertension, birth weight, type of delivery
Sagedal [35]	2017	Norway	591	296	295	2	Mod	24 w	Aerobic, strength training. Dietary counselling	Yes	60 min	-	Gestational weight gain, birth weight	Hypertension, gestational age, perineal tear
Silva-Jose [36]	2021	Spain	72	31	41	3	Mod	28 w	Aerobic, strength exercises, PFMT	Yes	55–60 min	80%	Hypertension	Gestational weight gain
Skow [37]	2021	Canada	59	31	28	3–4	50–70% Max HR	17 w	Aerobic exercises	No	25–40 min	-	Muscle sympathetic nerve activity	Gestational hypertension
Stafne [38]	2012	Norway	855	429	426	1	Mod–High	12 w	Aerobic, strength exercise	Yes	60 min	55%	Gestational diabetes	Hypertension
						2				No	45 min			
Stutzman [39]	2010	Canada	22	11	11	5	Low	16 w	Walking	No	30–50 min		Hypertension, heart rate variability	Baroreflex sensitivity
Taniguchi [40]	2016	Japan	118	60	58	3	Mod	6 + w	Brisk walking	Yes	30 min	80%	Duration of labor, type of delivery, birth weight	-
Tomic [41]	2013	Croatia	334	166	168	3	60–75% Max HR	28 w	Aerobic exercise	Yes	50 min	80%	Hypertension, birth weight, type of delivery	Gestational weight gain
Ussher [42]	2015	UK	785	392	393	2	Mod	6 w	Walking	Yes	30 min	70%	Smoking cessation, hypertension	Type of delivery, birth weight
Vázquez Lara [43]	2017	Spain	46	18	28	2	Mod	6 w	Aquatic activities	Yes	45 min	90%	Hypertension	Plasma volume
Yeo [44]	2010	USA	124	60	64	5	Mod	18 w	Stretching exercise	No	-	75%	Preeclampsia	Hypertension

Author (first author last name); year (year of study); country (country where the article was developed, usually the methods); type (type of article); N (total number of women analyzed); EG (number of women analyzed in the intervention group); CG (number of women analyzed in the control group); freq (weekly frequency of exercise sessions); intensity (moderate, high…); duration of program (program time, if the program has lasted 10 weeks, or if it has started in week 12 and ended in week 28, it was added as 16 weeks long); type of exercise (aerobic, muscle strengthening, etc.); superv. classes (whether or not there was supervision); duration of class (minutes of each session); adh. (adherence of the participants to the intervention in %). PFMT (pelvic floor muscle training).

## Data Availability

Data is unavailable due to ethical restrictions.
